# Metabolic Genes within Cyanophage Genomes: Implications for Diversity and Evolution

**DOI:** 10.3390/genes7100080

**Published:** 2016-09-29

**Authors:** E-Bin Gao, Youhua Huang, Degang Ning

**Affiliations:** 1 School of The Environment and Safety Engineering, Jiangsu University, No. 301, Xuefu Road, Zhenjiang 212013, Jiangsu Province, China; gaofei@ujs.edu.cn; 2 Key Laboratory of Tropical Marine Bio-resources and Ecology, South China Sea Institute of Oceanology, Chinese Academy of Sciences, No. 164, Xingangxi Road, Haizhu District, Guangzhou 5103401, Guangdong Province, China; 3 ACS Key Laboratory of Algae Biology, Institute of Hydrobiology, Chinese Academy of Sciences, No. 7, Donghu South Road, Wuchang District, Wuhan 430072, Hubei Province, China

**Keywords:** metabolism, cyanophage, genetic diversity, cyanobacteria, diagnostic marker

## Abstract

Cyanophages, a group of viruses specifically infecting cyanobacteria, are genetically diverse and extensively abundant in water environments. As a result of selective pressure, cyanophages often acquire a range of metabolic genes from host genomes. The host-derived genes make a significant contribution to the ecological success of cyanophages. In this review, we summarize the host-derived metabolic genes, as well as their origin and roles in cyanophage evolution and important host metabolic pathways, such as the light-dependent reactions of photosynthesis, the pentose phosphate pathway, nutrient acquisition and nucleotide biosynthesis. We also discuss the suitability of the host-derived metabolic genes as potential diagnostic markers for the detection of genetic diversity of cyanophages in natural environments.

## 1. Introduction

Viruses in general, and bacteriophages in particular, have been shown to be the most abundant biological entities on the planet, outnumbering bacteria by more than one order of magnitude [1,2]. Viruses are recognized as one of the major causes of microbial mortality in natural aquatic environments [3]. Through cell lysis, they exert a significant influence on microbial diversity and biogeochemical cycling [4,5,6]. Furthermore, viruses also impact the biological and ecological processes of host organisms through antagonistic co-evolution [7,8,9]. Some of the most extensively studied viruses are those that specifically infect cyanobacteria, called cyanophages. Cyanophages are of special interest due to their potential impact on cyanobacterial distribution, physiological processes and evolution [10,11,12,13,14,15,16].

Cyanophages have been shown to be a significant agent in regulating the dynamics and composition of cyanobacterial populations [17,18,19,20,21]. Like their host cyanobacteria, they are widespread and numerically abundant components of microbial communities in natural waters, and possess amazing amounts of genetic diversity and biological activity [22,23,24,25,26,27,28,29]. During the past decade, numerous cyanophages have been isolated from freshwater and marine environments. They morphologically belong to three different double-stranded DNA virus families; *Myoviridae*, *Podoviridae* and *Siphoviridae* [30,31,32,33]. On the basis of the sequencing of complete genomes, it has been found that cyanophages contain unique genetic resources [12,13,14,15,16,34,35,36,37,38,39,40,41,42], a vast majority of which are considered as genome database orphans. Previously, it was suggested that these unique genes probably encode specific enzymes or proteins for the cyanophage life cycle [43,44]. However, comparative genomic analyses have demonstrated that the majority of them show high similarity with cellular homologues of host cyanobacterial origin at the DNA sequence level [45,46]. There are many studies showing evidence of cyanophage acquisition of host genes encoding proteins or enzymes in key metabolic processes [47,48,49]. For example, many cyanophages infecting strains of marine unicellular cyanobacteria of the genera *Prochlorococcus* and *Synechococcus* carry copies of the *psbA* and *psbD* genes encoding the D1 and D2 proteins of photosystem II [48,49]. They have been observed to be transcribed and translated during infection and proposed to be involved in regulating the photosynthetic activities of the infected cyanobacterial cells [50,51]. Therefore, those genes linking cyanophages to the modulation of the host metabolic pathway are of particular interest because they likely contribute to overcoming biochemical bottlenecks in the metabolic processes of infected host cells [52,53,54,55].

Such genes encoding host homologues associated with metabolic processes have been termed auxiliary metabolic genes [54]. The occurrence of auxiliary metabolic genes in cyanophage genomes provides new insights into the novel interactions between cyanophages and their host cyanobacteria. In order to gain a better understanding of the significant implications of cyanophage-encoded metabolic genes, this review mainly focuses on the physiological and ecological roles of host-derived metabolic genes involved in key metabolic pathways, such as photosynthesis and pigment biosynthesis, phosphate metabolism, carbon metabolism and nucleic acid synthesis. Furthermore, we will discuss the possibility of using them as a diagnostic signature for analyzing the genetic diversity of cyanophage populations in water ecosystems.

## 2. Origin of Host-Derived Metabolic Genes

Viruses never cease their struggle against their respective hosts. For a long time, the interactions between viruses and their hosts have led to a reciprocal genome evolution. Under survival pressure, microbial hosts have evolved a variety of antiviral resistance mechanisms by acquiring exogenous nucleotide sequences from phages, which are incorpotated into host genomes, such as clustered regularly-interspaced short palindromic repeats (CRISPRs) [56,57]. On the other hand, viruses could acquire certain genes from their hosts during viral DNA synthesis to obtain a fitness advantage [58]. The acquisition of host genes and their integration in viral genomes makes a significant contribution to the evolution and diversity of viral populations.

A number of cultured cyanophage genomes have been completely sequenced [12,13,14,15,16,34,35,36,37,38,39,40,41,58,59], and revealed numerous DNA fragments of host-like metabolic gene origin, many of which encode important proteins for cellular and metabolic functions. There is increasing evidence that these host-like genes are frequently derived by phages through horizontal gene transfer from the viral gene pool or their hosts [39,60,61]. Through complete or partial genome sequencing, a variety of genes encoding homologues of host proteins has been found in many cyanophages [40,41,42,61]. A survey of cyanophages infecting marine cyanobacterium *Synechococcus* indicated that a copy of the *psbA* gene was found in more than 50% of cyanophage genomes isolated from the Red Sea [41]. Another survey screening for the presence of photosystem II core reaction center genes, *psbA* and *psbD*, revealed that 88% of cyanophage genomes possess *psbA* and 50% possess both *psbA* and *psbD*, and specifically, the *psbA* gene was found in all myoviruses [58]. It was suggested that the acquisition of photosynthetic genes by cyanophages is a widespread event. On the basis of cluster analysis, cyanophage-encoded *psbA* genes contain an identical 212-bp insertion that shows canonical characteristics of a group I self-splicing intron [41,62]; and the high light-inducible protein (HLIP) encoded by the cyanophage *hli* gene possesses a strongly conserved TGQIIPGF motif found in the C terminus of cyanobacterial homologues [13]. These genes, of cyanobacterial origin, were suggested to be derived multiple times from a potential host or by recombination with other cyanophages infecting similar hosts [49,58]. Based on phylogenetic trees, these host-derived genes cluster with those from their potential host cyanobacteria, implying that they originated from cyanobacteria and supporting the evidence that cyanophages acquired these genes horizontally from their cyanobacterial hosts multiple times [58]. Additionally, in some genomes of freshwater cyanophages, an *nblA* gene was found to encode a small polypeptide present in all organisms containing phycobilisomes [34,35,38]. The cyanophage NblA has a high amino acid identity with its homologues in cyanobacteria. It contains the highly conserved LTMEQ motif in the N terminus and two residues in the C terminus binding to the phycobilisome [34,35]. It was suggested that cyanophage *nblA* may have evolved by horizontal gene transfer from their host cyanobacteria.

The exchange via homologous recombination among phages, and between phages and their hosts, is also considered as one of the important processes for cyanophage evolution [50,53,59,63]. To improve their fitness, cyanophages have evolved a molecular mechanism for acquiring environmentally-significant genes of host origin. The acquisition of new genetic materials may contribute to the life cycle of cyanophages in specific environments and shape cyanophage genetic diversity. More importantly, only the most necessary genes for the adaptation of cyanophage to certain environmental conditions are expected to be enriched in cyanophage genomes. This may be related to the physical limitation on the phage genome, as well as the energetic demands of genome replication. Supporting this hypothesis, Thompson et al. showed that phage genes are generally shorter than their host homolog [54]. It was speculated that those host-derived metabolic genes may improve the physiological and ecological fitness of cyanophages by temporarily increasing host metabolic activities before cell lysis and also facilitate cyanophages’ adaptation to changing environments [64]. Furthermore, the host-derived metabolic genes carried by cyanophages are enabling a deeper understanding of the evolutionary mechanism of cyanophage genomes, and how they contribute to their genetic diversity.

## 3. Physiological Functions of Host-Derived Metabolic Genes

**Figure 1:**
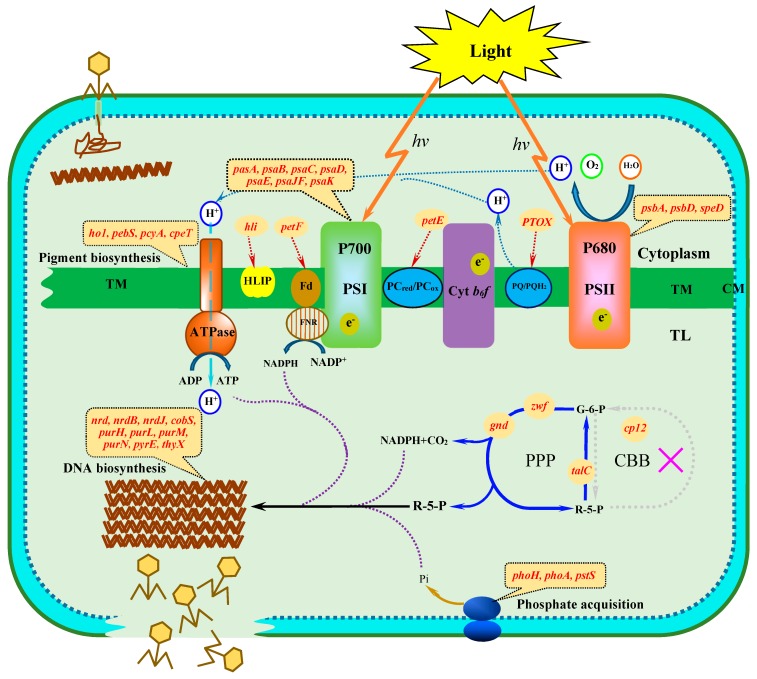
Model of the metabolic pathways regulated by cyanophage host-like genes in infected cyanobacterial cells. Cyanophage host-like genes are marked in red. Abbreviations: TM, thylakoid membrane; TL, thylakoid lumen; CM, cell membrane; PSI, photosystem I; PSII, photosystem II; PPP, pentose phosphate pathway; CBB, Calvin–Benson–Bassham; HLIP, high light-induced protein; PQ, plastoquinone (oxidized); PQH_2_, plastoquinone (reduced); PTOX, plastoquinol terminal oxidase; Cyt *b_6_f*, cytochrome *b_6_f* complex; PC_red_, plastocyanin (reduced); PC_ox_, plastocyanin (oxidized); FNR, ferredoxin-NADP reductase; Fd, ferredoxin; G-6-P, glucose-6-phosphate; R-5-P, ribose-5-phosphate.

Due to the lack of a metabolic system of their own, viral replication and structural assembly depend on host metabolic processes [65]. Host-derived metabolic genes are intimately related to the metabolism and life cycle of host cyanobacteria and may reflect the physiological interaction of cyanophages with their host cyanobacteria during infection. For example, Puxty et al. recently have shown that a cyanophage containing genes involved in carbon metabolism inhibits CO_2_ fixation during infection more rapidly than a cyanophage lacking them [52]. It has been suggested that the enzymes and proteins encoded by cyanophage metabolic genes exert a significant impact on the key metabolic processes during infection, such as the light-dependent reactions of photosynthesis, the pentose phosphate pathway (PPP), phosphate acquisition and DNA biosynthesis (Figure 1). More importantly, host-derived metabolic genes likely play a decisive role in the interaction between cyanophages and their cyanobacterial hosts and make a significant contribution to the unique selective pressure of cyanophages responding to ecological environments [66,67]. The host-derived metabolic genes may be the key genes for cyanobacterial growth and development, the expressed products of which can facilitate cyanophage genome replication and phage particle assembly. If a reaction, converting substrate to product, catalyzed by a host enzyme is limited during infection and if cyanophage replication depends on having enough product, the phage may encode its own enzyme to maintain the reaction and thus the availability of the product. This reaction could be limited because there is insufficient host enzyme, and therefore, cyanophages would increase the amount of the total enzyme; or because the activity of the host enzyme is suppressed under certain conditions, in which case the cyanophage enzyme would be active under those same conditions.

### 3.1. Photosynthetic Membrane

There are many studies showing that cyanophages carry genes related to two photosystems (PSI and PSII) of the cyanobacterial photosynthetic membrane and other photosynthesis-related genes involved in polyamine biosynthesis, photosynthetic electron transport and pigment biosynthesis [41,44,46,48,49,50,53]. The presence of photosynthesis genes in cyanophage genomes revealed that the life cycle of cyanophages is closely tied to the photosynthetic activity of host cyanobacteria [46]. It has been suggested that these genes involved in host cyanobacterial photosynthesis function so as to increase energy (ATP) and reductant (NADPH) for nucleotide biosynthesis and phage genome replication [52,54,66]. Among them are the genes *psbA* and *psbD* that are revealed to be most widespread in cultured and environmental cyanophage genomes [41,68]. Particularly, phage-encoded *psbA* gene expression has been detected during infection, yielding D1 proteins, and is suggested to play a functional role in photosynthesis for increasing phage fitness [50,52]. The decline of host D1 protein levels leads to the inhibition of photosynthetic activity [69,70], but cyanophage D1 proteins may help to supplement the content of host D1 proteins and maintain host photosynthesis. The homologue to polyamine biosynthesis gene *speD* was also found in marine cyanophage genome sequences [41]. The *speD* gene has been commonly recognized to catalyze the terminal step in polyamine synthesis and exerts a significant impact on the structure and oxygen evolution rate of the PSII reaction center [71]. If expressed, the cyanophage-encoded *speD* gene may serve to maintain the activity of the cyanobacterial PSII reaction center during phage infection. Furthermore, cyanophages possess a range of genes related to photosynthetic electron transport, such as plastocyanin (*petE*), plastoquinol terminal oxidase (PTOX) and ferredoxin (*petF*), for the redirection of the electron transport chain in the infected cyanobacterial cells [39,42,72]. Based on the homologies with cyanobacterial proteins, it was suggested that the cyanophage proteins participate in respiration and cyclic electron flow around PSI during infection, probably improving their fitness by increasing the production of ATP required for phage reproduction [73]. The cyanophage plastocyanin (*petE*) has been suggested to donate electrons to the alternate terminal electron acceptor cytochrome oxidase instead of PSI. PTOX is thought to accept electrons from reduced plastoquinone (PQH_2_) and to reduce O_2_ to H_2_O [74,75], which prevents oxidative damage. In addition, cyanophages frequently encode homologues of host genes involved in the biosynthesis of photosynthetic pigments, such as phycobilin (*hol*, *pebS*, *pcyA*) and phycoerythrin (*cpeT*) [76,77]. The cyanophage-encoded homologues of host genes involved in pigment biosynthesis have been proposed to regulate the consumption of the infected host carbon and photosynthetic energy. The potential functions of those genes have been revealed through their expression in heterologous cells and are possibly adjusted to the light conditions for increasing cyanophage replication [77].

To date, no genes related to PSI have been identified in all sequenced cyanophages. However, a cluster of genes forming a monomeric PSI were found in viral metagenomic sequences, which may have originated from cyanophages due to the presence of cyanophage-like genes on the scaffolds [44]. This cluster contains the genes *psaJF-C-A-B-K-D* and appears to encode a fusion protein between *psaJ* and *psaF*. Therefore, it was speculated that cyanophages may redirect metabolism from the respiratory chain through PSI to generate energy for replication.

### 3.2. Phycobilisome Degradation

Interestingly, the homologues of the *nblA* (non-bleaching) genes that were detected in freshwater cyanophage genomes may represent another example of the capture of photosynthesis system-associated genes from hosts [34,35]. The product encoded by the *nblA* gene is a small protein of cyanobacterial and red algae origin and plays an important role in the degradation of the major light-harvesting complex, phycobilisome, during nitrogen starvation. The *nblA*-like genes probably confer physiological and ecological benefits to cyanophages by reducing the absorption of excess light energy and providing an important source of amino acids for cyanophage protein synthesis through the degradation of the phycobilisome during infection.

### 3.3. Carbon Metabolism

Genes encoding key regulatory proteins or enzymes of the Calvin cycle-PPP of the carbon metabolism are also found in cyanophage genomes [42,54]. The cyanophage PPP-related genes mainly include the NADPH-producing enzyme glucose-6-phosphate dehydrogenase (*zwf*) and 6-phosphogluconate dehydrogenase (*gnd*), and the sugar transferase transaldolase (*talC*) [39]. Although no genes coding for the Calvin cycle proteins were detected, a gene for an inhibitor of the host cyanobacterial Calvin cycle, *cp12*, was identified in many cyanophage genomes [42]. It was suggested that cyanophages participate in modulating the PPP rather than the Calvin cycle of their hosts during infection. Indeed, there is recent evidence showing that a rapid shutdown of CO_2_ fixation was observed in marine cyanobacteria infected by cyanophages that contain genes modifying the central carbon metabolism (*cp12*, *talc*, *zwf*, *gnd*) [52]. The acquisition of PPP-related genes and *cp12* by cyanophages, contrasted with their absence from other viruses infecting non-cyanobacteria, suggests that they are a specific strategy used by cyanophages to redirect the host carbon metabolism during infection [42].

In cyanobacteria, the Calvin cycle fixes inorganic carbon to carbohydrates by consuming NADPH (electron carrier) and ATP (energy carrier) from the light reaction of photosynthesis during the day, while the PPP primarily oxidizes glucose at night to produce the nucleotide precursor ribose-5-phosphate (R-5-P) and the reducing equivalent (NADPH) [78]. The Calvin cycle and the PPP share several reactions, which reverse the direction of carbon metabolic flux. CP12 shuts down the Calvin cycle by forming a protein complex with two key Calvin cycle enzymes, PRK (phosphoribulokinase, *prkB*) and GAPDH (glyceraldehyde-3-phosphate dehydrogenase, *gap2*) [79], and then redirects the carbon flow toward the PPP. Thus, the presence of the *cp12* gene and PPP-related genes in cyanophage genomes was in support of the hypothesis that cyanophages may short-circuit the host carbon metabolism during infection and favor carbon flux through the PPP to generate NADPH and ribose-5-phosphate [42]. The NADPH and ribose-5-phosphate produced by the PPP and NADPH and ATP from the light reactions of photosynthesis, together, would make a significant contribution to the nucleotide biosynthesis that is essential for cyanophage replication [54].

### 3.4. Phosphate Acquisition

Phosphorus is an essential element for nucleotide biosynthesis and DNA replication, but extremely scarce in oligotrophic waters and thought to be one of the limiting factors for cyanobacterial growth [80,81,82]. Thus, it is not surprising that some phosphorus-acquisition genes, such as phosphate-inducible genes *pstS* and *phoH* and alkaline phosphatase gene *phoA,* which are regulated by the PhoR/PhoB two-component regulatory system to sense phosphorus availability, were found in the genomes of cyanophages infecting cyanobacteria [42,66,83]. These genes could be upregulated in response to phosphate starvation in host cells and play an important role in regulating phosphorus absorption and the transportation of host cells under low-phosphorus content or phosphorus-deprived conditions. The *pstS* gene encoding for a periplasmic high-affinity phosphate-binding transporter has been detected in several cyanophages [82], which were isolated from low-phosphorus oligotrophic waters. This suggests that cyanophages probably use the *pstS* gene that they encode to enhance the infection cycle through increasing phosphate availability in the infected host cells [84]. Cyanophage-encoded phoA lacks similarity to bacterial phoA based on sequence analysis, and its function is not clear. However, the alkaline phosphatase gene, *phoA*, encoded by cyanophages, may be related to the acquisition of organic phosphorus from the environment or from within the host, which contributes to cyanophage replication under the conditions of phosphorus depletion [66,82]. The phosphate-inducible gene *phoH* was found to be the most widespread phosphorus regulon detected in marine and freshwater cyanophage genomes [63,85], but its function has not been experimentally confirmed. Based on bioinformatic analyses, it is suggested that *phoH* genes may be part of a multi-gene family with divergent functions from phospholipid metabolism and RNA modification to fatty acid beta-oxidation [86]. These phosphorus-associated genes are in support of the hypothesis that there is a selective pressure for cyanophages to acquire environmentally-significant genes that might serve to power phosphorus acquisition during the infection of phosphate-starved cells. Furthermore, due to the high demand on a significant amount of phosphorus for the replication of cyanophage genomes, they could confer a fitness advantage to cyanophages by influencing host phosphorus acquisition in phosphorus-limited conditions.

### 3.5. DNA Biosynthesis

Several genes involved in the nucleotide biosynthesis pathway have been discovered in all known T4-like cyanophage genomes. Of these, the most prevalent gene is ribonucleotide reductase (RNR), which can provide the building blocks for DNA synthesis through reducing ribonucleotide diphosphates to deoxyribonucleotide diphosphates during the nucleotide metabolism [87]. This suggests that cyanophages could use RNRs that they encode to degrade host DNA for the genome replication of progeny phages. These RNRs are commonly found in lytic T4-like phages, but are not common in lysogenic phages. These nucleotide biosynthesis genes are thought to be essential for the rapid replication of lytic phages [12]. The rate of DNA synthesis of phages encoding RNR was found to be 10 times higher than that of phages lacking these enzymes [88]. A cobalt chelatase subunit *cobS* gene, which catalyzes the final step in cobalamin biosynthesis and could be potentially associated with RNR, was found in many T4-like cyanophage genomes. Cobalamin is an important cofactor of cyanobacterial class II RNRs during nucleotide metabolism [89] and makes a significant contribution to increasing the activity of RNRs for DNA biosynthesis. It is becoming evident that the cyanophage gene *cobS* is not actually homologous of bacterial *cobS*. However, if the functions of *cobS*, found in cyanophages, were similar to those of host homologs, it is tempting to speculate that the cyanophage-encoded *cobS* gene contributes to enhancing the production of cobalamin in the infected host cells and then provides physiological benefits for cyanophage genome replication.

Additionally, cyanophages encoded several genes related to purine biosynthesis, including *purH*, *purl*, *purM* and *purN*, and pyrimidine biosynthesis, such as *pyrE* and *thyX* [41]. With the help of different purine and pyrimidine biosynthesis enzymes, cyanophages catalyze many important phases of DNA biosynthesis to provide a great amount of deoxynucleotide triphosphates for their DNA replication and biosynthesis. Among these genes, the *thyX* gene is found in all three types of cyanophages and is thought to be a critical and limiting enzyme for DNA biosynthesis and modification [67]. The acquisition of DNA metabolism genes may be an ecologically-selective mechanism for cyanophages living in stress environments. Although the degradation and reuse of host DNA is very important for DNA biosynthesis of the progeny cyanophages, it is believed that de novo DNA synthesis, catalyzed by cyanophage-encoded nucleotide biosynthesis genes, is likely critical for cyanophages to achieve the optimal production [50,64].

## 4. Signature Markers for Cyanophage Evolution

The high abundance of viruses in natural waters has attracted particular attention to the understanding of their evolution and genetic diversity over the past decade. Due to the lack of a single universal gene, such as bacterial 16S rRNA, common to all viruses for PCR-based surveys, assessing the genetic diversity of the total viral community in natural water environments is challenging. Cyanophages are a group of extremely diverse tailed viruses. Although a variety of structural genes, such as *g20* (portal protein) [90,91,92,93,94], *mcp* (major capsid protein) [95], *g91* (tail sheath protein) [96], *Syn_g101* (putative tail fiber) [63] and DNA polymerase [97,98], were used as marker molecules for investigating the genetic diversity and phylogenetic relationship of cyanophages, they are found to be restricted to specific groups that can be used as PCR targets. For example, the DNA polymerase gene is restricted to only a subset of the T7-like phage group, and the structural gene (*g20*) that encodes a portal protein in myophage has been restricted to T4-like cyanophages. Although these results revealed the high diversity of cyanophages in natural waters, the primers not only target cyanomyophages, but also other myoviruses that do not infect cyanobacteria.Hence, finding common marker genes is essential for studying the genetic diversity and phylogenetic relationship of cyanophages.

Host-derived metabolic genes are commonly found in cyanophages, but not other phages infecting non-cyanobacteria and, consequently, provide ideal molecular markers to investigate the genetic diversity and evolutionary history of cyanophages in nature [99]. In contrast to viral structural genes, the use of host-like metabolic genes as candidate marker genes would enable a deeper understanding of the ecological relationship and molecular diversity of cyanophages in response to specific environments. Currently, a range of host-derived metabolic genes have been used as signature biomarkers to investigate the genetic diversity and the evolutionary relationship of cyanophages from cultured and environmental samples (Table 1). The cyanophage-encoded photosynthesis genes *psbA* and *psbD* have been used as additional marker molecules to investigate the genetic diversity and evolution of phage populations in natural aquatic environments [100,101,102,103,104]. Phylogenetic analyses, based on the *psbA* gene, indicated that cyanophages from freshwater environments possess an evolutionary history different from their marine counterparts. It was suggested that *psbA* has the potential to address greater insights into the population structure, phylogenetic relationship and genetic diversity of cyanophage communities in marine and freshwater environments and of their infecting host cyanobacteria. The *psbA* gene has been found to be widespread in cyanomyoviruses and cyanopodoviruses [47,48], and is considered a more complete marker than *g20* for cyanophages. However, due to the lack of this signature gene in the cyanosiphoviruses, and the occurrence of introns within cyanophage *psbA* genes, the primers targeted cyanophage *psbA* sequences, usually leading to underestimated diversity [62]. Additionally, the global distribution of cyanophages has been identified through examining the presence of homologues of MazG, a regulator of programmed cell death in *Escherichia coli*, in cyanophages isolated from different oceanic regions [105,106]. Based on the phylogenetic tree of the *mazG* gene from phages and bacteria, it was suggested that cyanophages cannot acquire the *mazG* genes from their primary hosts, and the frequent gene exchange may occur among different marine cyanophages by horizontal gene transfer [85,107]. More recently documented, *phoH* has been developed as a novel signature gene to assess the genetic diversity of viruses in multiple families of double-stranded DNA tailed phages [108]. Through the deep sequencing of the *phoH* gene, the composition and diversity patterns of the phages in the Sargasso Sea were shown to be closely associated with spatio–temporal structure [109]. Also, *phoH* has been found in many cyanophages infecting cyanobacteria, such as marine cyanophages P-SSM2, P-SSM4 [41], Syn9 [15], and freshwater cyanophages Ma-LMM01 [34] and MaMV-DC [38]. From the analysis of the *phoH* phylogenetic tree, a high diversity of cyanophages has been found in all sampling sites [108]. However, due to the presence of the *phoH* gene in phages infecting autotrophs and heterotrophs, caution is needed to use this signature gene because it could lead to an increased signal in cyanophage diversity in a chosen environment.

If a single metabolic gene was used as a diagnostic marker, information about the genetic diversity would be restricted to a specific group of cyanophages that carry the marker metabolic gene. For better investigation of the diversity and evolutionary history of cyanophages in a relatively comprehensive way, it is essential to combine a variety of host-derived metabolic genes encoded by cyanophages or identified from certain environments to characterize their gene type and composition by using microbial ecological techniques, such as metagenomics and metaproteomics.

## 5. Future Perspectives

Since the isolation of the first cyanophage from natural waters, the biological function and ecological importance of cyanophages in aquatic ecosystems have received great attention. Particularly, the potential roles of cyanophages in terminating harmful cyanobacterial blooms have become one of the important focuses of environmental science and virology. In recent years, studies of the genetic diversity and evolutionary history of cyanophages have advanced through the use of various methods of molecular biology [110,111]. However, information about the ecological impact of cyanophages on cyanobacterial populations in natural environments is still limited; many studies should be further performed to address the molecular and physiological mechanism of cyanophage–cyanobacterial interaction during infection. Furthermore, due to the limitation of microbial culture techniques, it is still difficult to select unknown cyanobacterial hosts for the isolation and cultivation of a novel cyanophage in the lab and, thus, the study of the physiological relationship between cyanophages and their host cyanobacteria.

There is no doubt that the ecological functions of cyanophages in natural aquatic environments are much more important than what we have known from cultivated cyanophages. The genetic diversity of cyanophages in natural environments is still largely unclear. Nevertheless, the discovery of metabolic genes in sequenced cyanophages provides us with a clue for using the cyanophage metabolic genes as signature markers to elucidate the physiological and ecological characteristics of cyanophages in natural environments. For future studies on the genetic diversity and ecological roles of cyanophages, traditional culture methods of viral isolation and purification should be further improved to establish different systems for cultivating more cyanophages from natural waters and to analyze their genome sequences for evaluating the possible presence and biological functions of auxiliary metabolic genes. A variety of technical means, such as isotopic labeling, physiology experiments, mutagenesis, structural biology, enzymology and gene knockout, would be comprehensively applied to determine the potential roles of host-derived metabolic genes during the interaction of cyanophages with their host cyanobacteria. On the other hand, it is essential to adopt modern molecular biological techniques, such as viral shotgun metagenomics, gene chip and proteomics, to identify different cyanophage metabolic genes from marine and freshwater environments, and subsequently use them as markers to characterize the population dynamics and genetic diversity of cyanophages in the same locations. Therefore, given the origin and biological functions of host-derived metabolic genes encoded by cyanophages, their utility as signature genes for cyanophages will serve to illustrate the phylogenetic relationship and population structure of cyanophages in natural environments and also address the physiological mechanism of cyanophage–cyanobacterial interaction from the genetic and metabolic level.

Currently, PCR-based diagnostics are considered to be a much more effective method to assess the genetic diversity of cyanophages from multiple samples, time points and locations. As more cyanophage genome or metagenomics data sets become available, it is possible that the appropriate PCR primers could be designed to capture existing signature genes, including metabolic genes and structural genes, which will increase coverage for the true diversity of cyanophage populations in chosen habitats. With the decline of genome sequencing costs, more metabolic genes would be identified from the cyanophage genomes and metagenomics, and become the best signature in combination with PCR-based fingerprinting to investigate cyanophage diversity and evolution. This will become the ideal way for studying the evolutionary and ecological roles of cyanophage population in a wide range of environments.

## Figures and Tables

**Table 1:** Primer list of host-derived metabolic genes described in this study.

Signature Gene	Function	Primer Sequence	References
*psbA*	Photosynthesis protein D1	psbA-F: 5′-GTNGAYATHGAYGGNATHMGNGARCC-3′ psbA-R: 5′-GGRAARTTRTGNGCRTTNCKYTCRTGCAT-3′	[100]
		Pro-psbA-F: 5′-AACATCATYTCWGGTGCWGT-3′ Pro-psbA-R: 5′-TCGTGCATTACTTCCATACC-3′	[58]
		psbA-93F: 5′-TAYCCNATYTGGGAAGC-3′ psbA-341R: 5′-TCRAGDGGGAARTTRTG-3′	[109]
*psbD*	Photosynthesis protein D2	psbD-26Fa: 5′-TTYGTNTTYRTNGGNTGGAGYGG-3′ psbD-26Fb: 5′-TTYGTNTTYRTNGGNTGGTCNGG-3′	[58]
		psbD-54Fa: 5′-GTNACNAGYTGGTAYACNCAYGG-3′ psbD-54Fb: 5′-GTNACNTCNTGGTAYACNCAYGG-3′	[58]
		psbD-308Ra: 5′-YTCYTGNGANACRAARTCRTANGC-3′ psbD-308Rb: 5′-YTCYTGRCTNACRAARTCRTANGC-3′	[58]
		psbD-F: 5′-GGNTTYATGCTNMGNCARTT-3′ psbD-R: 5′-CKRTTNGCNGTVAYCAT-3′	[27]
*cobS*	Putative porphyrin biosynthetic protein	cobS-For: 5′-BACYGTWTGGCACAAYGG-3′ cobS-Rev: 5′-CTTRGTNTCMTCATCRAARCG-3′	[63]
*mazG*	Nucleoside triphosphate pyrophosphohydrolase	mazG-For: 5′-CTTCTTACTGCTGSYGTTGG-3′ mazG-Rev: 5′-TTATCKGTCRTCKRCWGATT-3′	[104]
*phoH*	Putative phosphate protein	vPhoHf: 5′-TGCRGGWACAGGTAARACAT-3′ vPhoHr: 5′-TCRCCRCAGAAAAYMATTTT-3′	[106]
		phoH-For: 5′-GARATYGGDTTCYTDCCTGG-3′ phoH-Rev: 5′-ACWARWCCAGADCKWACRATRTC-3′	[63]
